# Phytoplankton and Eutrophication Degree Assessment of Baiyangdian Lake Wetland, China

**DOI:** 10.1155/2013/436965

**Published:** 2013-07-25

**Authors:** Xing Wang, Yu Wang, Lusan Liu, Jianmin Shu, Yanzhong Zhu, Juan Zhou

**Affiliations:** ^1^State Key Laboratory of Environmental Criteria and Risk Assessment, Chinese Research Academy of Environmental Sciences, Beijing 100012, China; ^2^State Environmental Protection Key Laboratory of Estuarine and Coastal Environment, Chinese Research Academy of Environmental Sciences, Beijing 100012, China

## Abstract

Eight typical sampling sites were chosen to investigate the phytoplankton community structure and to assess the eutrophication degree of Baiyangdian Lake in 2009. Our results showed that among the total 133 species identified, Cyanophyta, Chlorophyta, and Bacillariophyta dominated the phytoplankton community. In spring, Chlorophyta and Bacillariophyta were the dominant phyla, and the dominant species included *Chlorella* sp., *Chroomonas acuta* Uterm., and *Microcystis incerta* Lemm.; the density of the phytoplankton ranged from 496 × 10^4^ to 6256 × 10^4^ cells/L with an average of 2384 × 10^4^ cells/L. However, Chlorophyta and Cyanophyta became the dominant phyla in summer, and the dominant species were *Chlorella* sp., *Leptolyngbya valderiana* Anagn., and *Nephrocytium agardhianum* Nageli.; the density of the phytoplankton varied from 318 × 10^4^ to 4630 × 10^4^ cells/L with an average of 1785 × 10^4^ cells/L. The density of the phytoplankton has increased significantly compared to the previous investigations in 2005. The index of Carlson nutritional status (TSIM) and the dominant genus assessment indicated that the majority of Baiyangdian Lake was in eutrophic state.

## 1. Introduction

Eutrophication is a phenomenon, in which the excess trophic substances (i.e., nitrogen and phosphorus) in lakes, reservoirs, estuaries, rivers, and certain coastal waters cause a great increase in algae and a decrease in dissolved oxygen, thus, leading to serious death of a lot of fishes and other hydrophytes. A lot of freshwater water bodies have occurred eutrophication in the 1990s [1], and it was the first time for eutrophication began to become the major pollution problem of lakes and reservoirs in most countries such as Europe and North America. An investigation showed that eutrophication took place in 54% of Asian lakes, 53% of European lakes, 48% of lakes in North America, 41% of lakes in South America, and even 28% of lakes in Africa [2]. For example, Japan's second largest lake (Lake Kasumigaura) once played a very important role in irrigation, human life, industry, inland fishery, recreation, and so on; however, serious eutrophication has been observed in this lake since the early 1900, and this once well-known tourist attraction was forced to shut down due to the deteriorative water quality [3].

There are in total 2,759 lakes with the area of more than 1 km^2^ in China, which covers an area of 91019 km^2^ and makes up 0.95% of land area in China. About one-third of these lakes is fresh water lake, and they are mainly located in eastern coastal area and the lower-middle reaches of Yangtze River. In recent years, the rapid development of economy, the inappropriate development and utilization of water resources, and the worsening agriculture nonpoint pollution have resulted in serious eutrophication: most of these lakes have been plagued by eutrophication or are in the course of eutrophication development [4]; the structure and function of the lake ecosystems have degenerated with the frequent occurrence of blue-green algae blooms and pollution-induced water shortage. These water problems seriously affect the production activities and life of people in the lake region, limit the sustainable development of regional social economy, and cause great financial losses and social problems [5].

Phytoplankton including many species is widely distributed in the aquatic ecosystem, which maintains the structural functions of ecosystem and plays an important and irreplaceable role of indicator and purifier on lake pollution, through participating in material cycle and energy flow in lakes [6, 7]. In recent years, the species and community structure characteristics of phytoplankton combined with the chemical detection of water quality have been generally accepted as environmental assessment indicators [8]. Among them, the index of Carlson nutritional status (TSIM) and the dominant genus assessment have been widely applied for the assessment of eutrophication in lakes and reservoirs [9, 10].

Baiyangdian Lake, located in the eastern part of North China Plain, is the largest fresh water lake in Haihe River Basin. It is also the largest inland shallow lake in North China and is historically given the name of “the Pearl of North China” because of its excellent water quality and great biodiversity. However, in the highly populated Baiyangdian Lake area, industrial effluents and domestic sewage flow directly into the lake through surface runoff [11], upstream water supply declines, and water level drops, which results in low self-purifying capacity of this lake. As a result, the water quality of this lake is increasingly more worse [12] with serious eutrophication [13].

The objectives of this paper were to identify the species composition and spatial distribution of the phytoplankton and to evaluate the eutrophication degree in Baiyangdian Lake.

## 2. Materials and Methods

### 2.1. Study Area

Baiyangdian Lake, with an area of about 366 km^2^, is a typical plant-dominated shallow freshwater lake consisting of about 143 lake parks, with a maximum depth of 4.0 m. Reeds and cattails are the dominant macrophyte, covering about 22% of the lake area. Raised fields with reeds and cattails coverings are divided into multiple blocks by more than 3,700 artificial ditches. These ditches are regarded as multiple connected corridors in the reed wetland landscape. It has the semiarid monsoon climate with the average annual precipitation of 570.2 mm, and the average annual evaporation in this region is 1369 mm, which is far higher than the average annual precipitation [14]. Meanwhile, the reduction of lake surface area and depth worsens the eutrophic situation.

### 2.2. Sample Collection and Analysis

**Figure 1:**
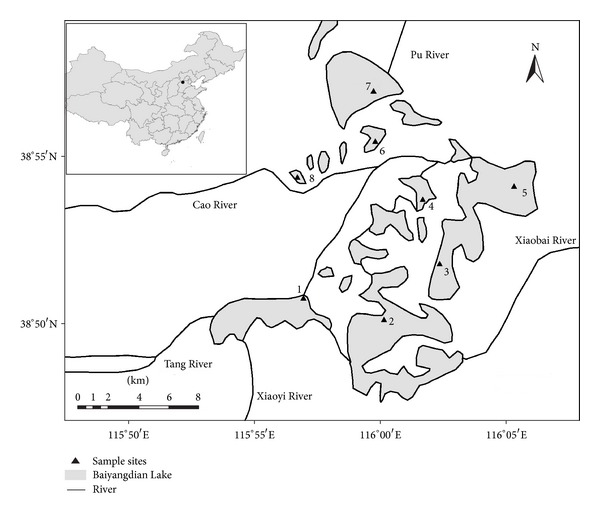
Sampling sites of the Baiyangdian Lake in Hebei Province, China.

Eight sampling sites were chosen in the Baiyangdian Lake (115°56′ to 116°06′E, 38°49′ to 38°57′N; Figure 1), and phytoplankton and relevant environment factors were investigated in the early April (spring) and in the middle of June (summer) of 2009. Quantitative sample collection of phytoplankton is as follows: organic glass hydrophore was used to collect water samples; the volume of water sample was 1000 mL; for water which was no more than 3 meters deep and the water masses of which were well mixed, one sample of 1000 mL needed to be collected from the surface (0.5 meter), while for water which was 3 to 10 meters deep, one sample of 500 mL from the surface and one sample of 500 mL from the bottom needed to be collected and mixed. Lugol's solution was used to fix samples, and 1% (vol) formalin solution was used to preserve samples. The treatment, analysis method, and water quality analysis method of the phytoplankton samples were carried out according to the standard methods from *Lake Ecosystem Observation Method* [15].

### 2.3. Data Processing

Data were proceeded using SPSS and PRIMER V6 software packages [16] to get ecological indicators such as species number, density, Shannon-Weiner index, and richness index of phytoplankton.

Shannon-Weiner index is calculated using formula (1):

(1) $$\begin{array}{c} H^{\prime }=-\sum_{i=1}^{s}P_{i}\ln P_{i}, \end{array}$$

where *P*_*i*_ represents the percentage of species *i* in the samples; for example, if the total number of species is *N* and the number of species *i* is *n*_*i*_, then *P*_*i*_ = *n*_*i*_/*N*.

Richness index is calculated using formula (2):

(2) $$\begin{array}{c} d=\frac{\left(S-1\right)}{\log_{2}M}, \end{array}$$

where *S* is the number of species in the samples collected from certain sampling sites and *M* is the number of individuals of all the species in this sampling site.

Uniformity is calculated using formula (3):

(3) $$\begin{array}{c} J^{\prime }=\frac{H^{\prime }}{\ln S}, \end{array}$$

where *H*′ is the Shannon-Weiner index and *S* is the number of species in the samples collected from certain sampling sites.

## 3. Results and Discussion

### 3.1. Community Composition and Biodiversity

**Figure 2:**
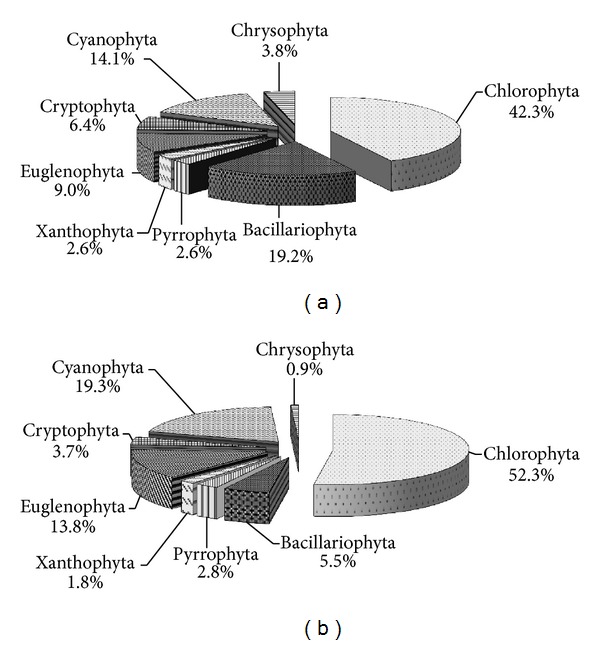
Composition percentage of the main phytoplankton in the study area in spring (a) and summer (b).

All the phytoplankton collected in both dates could be categorized into 8 phyla and 133 species (genus). Amongst them, 8 phyla and 78 species (genus) were observed in spring, and Chlorophyta, Cyanophyta, and Bacillariophyta were the dominate phytoplankton community with the greatest number of Chlorophyta including 33 species (genus), which accounted for 42.3% of the total number of algae. Fifteen species (genus) of Bacillariophyta and 11 species (genus) of Cyanophyta were observed, accounting for 19.2% and 14.1% of the total number of algae, respectively. There were 7 species (genus) for Euglenophyta and 5 species (genus) for Cryptophyta, accounting for 9.0% and 6.4% of the total number of algae, respectively. However, there were only 2 species (genus) for Pyrrophyta, 2 species (genus) for Xanthophyta, and 3 species (genus) for Chrysophyta, accounting for 2.6%, 2.6%, and 3.8% of the total number of algae, respectively, (Figure 2(a)).

Eight phyla and 133 species (genus) were observed in summer, and Chlorophyta, Cyanophyta, and Euglenophyta were the dominate phytoplankton community with the greatest number of Chlorophyta including 57 species (genus), which accounted for 52.3% of the total number of algae. Twenty-one species (genus) of Cyanophyta and 15 species (genus) of Euglenophyta were observed, accounting for 19.3% and 13.8% of the total number of algae, respectively. There were 6 species (genus) for Bacillariophyta and 3 species (genus) for Pyrrophyta, accounting for 5.5% and 2.8% of the total number of algae, respectively. However, there were only 2 species (genus) for Xanthophyta, 4 species (genus) for Cryptophyta, and 1 species (genus) for Chrysophyta, accounting for 1.8%, 3.7%, and 0.9% of the total number of algae, respectively, (Figure 2(b)). In spring, the first dominant species in Baiyangdian Lake is *Chlorella sp.* which belongs to the Chlorophyta phylum with the occurrence frequency of 100%. The second dominant species are *Chroomonas acuta *Uterm. and *Microcystis incerta *Lemm., which belong to the Cryptophyta and Cyanophyta phyla with the occurrence frequency of 87.5% and 100%, respectively. In summer, the first dominant species in Baiyangdian Lake is *Chlorella sp.* which belongs to the Chlorophyta phylum with the occurrence frequency of 100%. The second dominant species are *Leptolyngbya valderiana *Anagn. and *Nephrocytium agardhianum *Nageli. which belongs to the Cyanophyta and Chlorophyta phyla with occurrence frequency of 70% and 88%, respectively.

**Figure 3:**
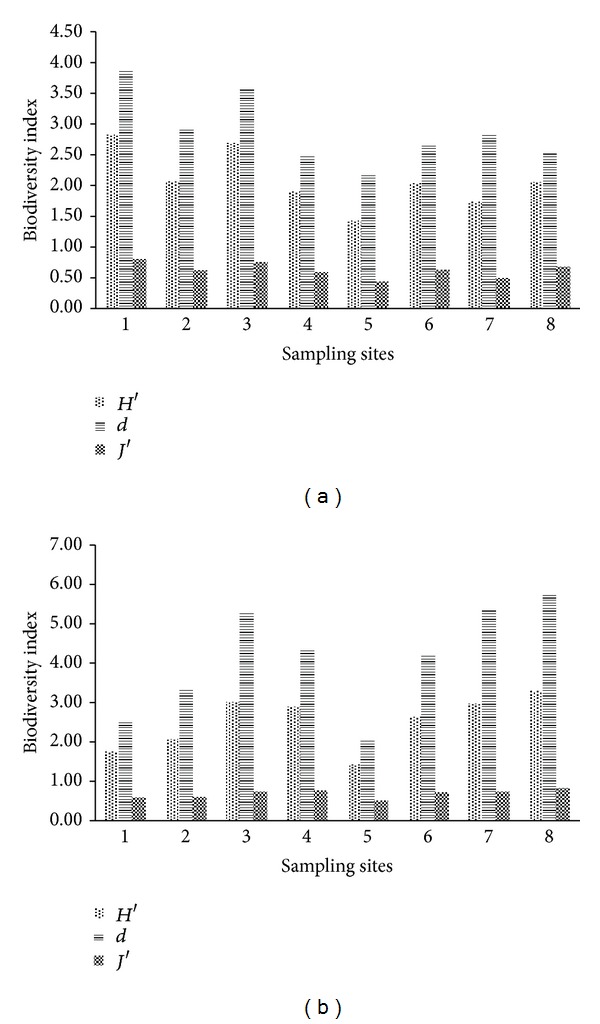
Biodiversity index of the phytoplankton in different sampling sites in Baiyangdian Lake in spring (a) and summer (b).

Figure 3 shows the biodiversity index of the phytoplankton in Baiyangdian Lake sampling sites in spring (a) and summer (b). As shown in Figure 3(a), the Shannon-Weiner index of phytoplankton was 1.43~2.82 with an average of 2.09, and the species richness index of phytoplankton was 2.15~3.89 with an average of 2.88. The Shannon-Weiner index for Site 5 was the lowest (1.43), and the richness index is 2.15; the Shannon-Weiner index for Site 1 was the highest (2.82), and the richness index is 3.89. It is obvious that the space distribution tendency of Shannon-Weiner index and species richness index was consistent. The uniformity index was 0.44~0.80 with an average of 0.62. In summer, the Shannon-Weiner index of phytoplankton was 1.42~3.29 with an average of 2.50, and the species richness index of phytoplankton was 2.05~5.78 with an average of 4.11 (Figure 3(b)). The Shannon-Weiner index for Site 5 was the lowest (1.42), and the richness index is 2.05; the Shannon-Weiner index for Site 8 was the highest (3.29), and the richness index is 5.78. The space distribution tendency of Shannon-Weiner index and species richness index was consistent. The uniformity index of phytoplankton was 0.51~0.82 with an average of 0.68. According to this, in Baiyangdian Lake, there were more species of phytoplankton in summer than in spring but there was no dominant group; Chlorophyta and Cyanophyta dominated the community in both spring and summer. In sampling sites close to residential area, fish culturing cages, livestock, and poultry farms which were under great influence of human activities, there were more varieties of phytoplankton; while in sampling sites, which were situated in lake outlets and in standing water, there were fewer varieties of phytoplankton. This is consistent with the results in previous studies of phytoplankton in Baiyangdian Lake [17].

### 3.2. Phytoplankton Density

**Figure 4:**
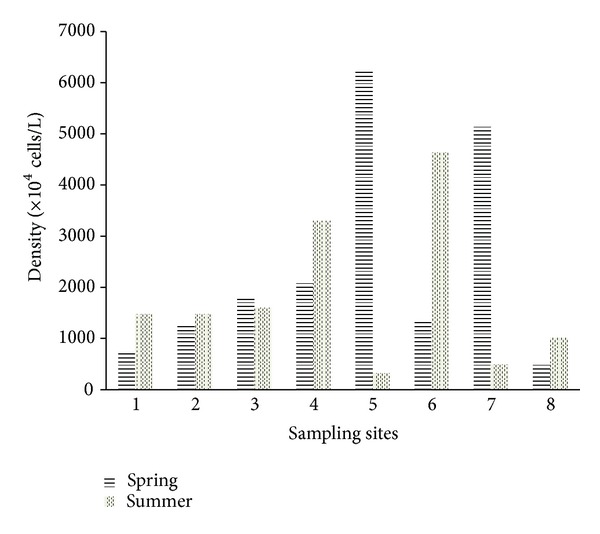
Comparison of phytoplankton density in different sampling sites in Baiyangdian Lake in spring and summer.

The number of phytoplankton in Baiyangdian Lake varied greatly between spring and summer. In spring, the density of phytoplankton varied from 496 × 10^4^ to 6256 × 10^4^ cells/L with an average of 2384 × 10^4^ cells/L; the density varied greatly between different sampling sites: the density of phytoplankton cells in Sites 5 and 7 was high, while the density of Sites 1 and 8 was low. In summer, the density of phytoplankton varied from 318 × 10^4^ to 4630 × 10^4^ cells/L with an average of 1785 × 10^4^ cells/L (Figure 4); the density varied greatly between different sampling sites: the density of phytoplankton cells in Sites 4 and 6 was high, while the density of Sites 5 and 7 was low. Generally speaking, the density of phytoplankton and dominant species can indicate the eutrophication degree of certain water. As species of high pollution tolerant, if Cyanophyta increases sharply and finally becomes the dominant species, it indicates that the water is eutrophic, that is to say, the higher the density of Cyanophyta cells is, the more serious the eutrophication is [18]. According to this survey, cyanophyta and Chlorophyta became dominant in the phytoplankton community, that may be caused by the increased organic matters after organic matters in industrial wastewater and domestic sewage came into Baiyangdian Lake [19] and resulted in the increase of varieties and number of phytoplankton, especially these species with high pollution tolerance.

To get a general understanding of the change of phytoplankton community in Baiyangdian Lake, data of the three times surveys of phytoplankton since 2005 were compared (Table 1), and dynamic variations of phytoplankton in Baiyangdian Lake in recent years were analyzed from the aspects of species composition, density, and dominant species. The data of the years 2005 and 2006 were averages from the 8 sampling sites in Baiyangdian Lake in spring and summer, and the data of the year 2009 were averages from the current survey in 8 sampling sites in Baiyangdian Lake in spring and summer. The survey methods were the same.

Table 1 shows the variations of phytoplankton density and dominant species in Baiyangdian Lake in recent years. Taking the year 2005 as the reference point, the average phytoplankton density decreased 0.22 times in 2006, whereas the average phytoplankton density increased 2.41 times in 2009. In the three surveys, Chlorophyta and Cyanophyta were the mainly dominant species. The annual variations tendency of different algae phylums were different; the phytoplankton variety number tended to decrease, while the phytoplankton density tended to increase. In the current survey, the phytoplankton density increased observably, because the nutrient load in Baiyangdian Lake changed; when sampling, algae were growing and blooming, and as the temperature became higher and the light became stronger, the water environment became more favorable for the growth of phytoplankton which abundance increased as a result. Generally speaking, in mesotrophic lakes, Pyrrophyta, Cryptophyta, and Bacillariophyta are the dominant species, and in eutrophic lakes, Chlorophyta and Cyanophyta are the dominant species [21]. From this, a conclusion can be drawn that *Chlorella sp. *and *Microcystis incerta *Lemm. which belong to the Chlorophyta and Cyanophyta phyla, respectively, are becoming the dominant species, indicating that Baiyangdian Lake had become eutrophic and organic matters tended to increase year by year.

### 3.3. Relationship between Phytoplankton and Environmental Factors

Nine environment factors, chemical oxygen demand (COD), potassium permanganate index (COD_Mn_), total nitrogen (TN), total phosphorus (TP), chlorophyll a (Chla), pH, dissolved oxygen (DO), secchi depth (SD), and temperature, were selected in the eight sampling sites. The Pearson correlation analysis showed that no significant correlation was observed between phytoplankton cell density and environmental factors in spring (Table 2). In summer (Table 3), phytoplankton cell density was greatly influenced by DO (*r* = 0.813, *P* = 0.014), Chla (*r* = 0.818, *P* = 0.024), and TP (*r* = 0.833, *P* = 0.010), and there was a positive correlation between them. According to this, the best environment factor combination could not be identified which influenced the space distribution of phytoplankton in Baiyangdian Lake in summer, and DO, Chla, and TP were probably the important factors which influenced the space distribution of phytoplankton in Baiyangdian Lake in summer. The main reason for this is that summer is usually the time for algal bloom occurrence in eutrophic lakes. In the sampling Sites 3 and 7, because breeding industry and crop farming were flourishing, the concentration of nutrients in water was high, while algae bloom, DO, and the phytoplankton cell density were low. In the sampling Sites 5 and 1, because the water was open and far from pollution source, the water quality was good and the phytoplankton cell density was low.

Phytoplankton cell density varied greatly from season to season mainly due to the water temperature. Environment factors have different influences on the community structure and species number of phytoplankton in different lakes: SONG Xiao-lan's study on the community structure of phytoplankton in Taihu Lake and Wulihe River found that wind wave and eutrophication degree were the restrictive conditions for growth of phytoplankton species [22]. Jeppesen pointed out that in lakes the density of filtering-feeding fish was a factor closely correlated with the variety and number of phytoplankton [23]. However, other unknown physical, chemical, and biological factors can probably directly or indirectly influence the number of phytoplankton in a certain way, because the growth of phytoplankton is also related to many other factors (e.g., water stability, climate, lake area, lake depth, spatial distribution of organic matter and heavy metals in wetland soils, the community structure, and density of hydrophyte) [24–35]. Therefore, it is difficult to do “dose-effect analysis” of the interaction between the number of phytoplankton and the abovementioned factors. It is suggested that in the future research, more efforts should be made to study lake type, climate characteristics, and surrounding environment, and simulated experiments should be also included to the impact factors on variation of phytoplankton number.

### 3.4. Eutrophication Degree Assessment

Generally, the excessive growth of phytoplankton is the characterization of eutrophication. Chla, SD, and dominant species are usually regarded as the most important indicators for the assessment of eutrophication degree. In this paper, the index of TSIM and the dominant genus assessment were used to assess the trophic status of Baiyangdian Lake [36]. The index of Carlson nutritional status (TSIM) can elaborately describe the change of water trophic status and can also improve water quality monitoring and assessment. The method is to grade the lake trophic status with numbers from 0 to 100 according to the relation between SD, Chla, and TP. Index under 30 indicates oligotrophic water, index from 30 to 50 indicates mesotrophic water, and index from 50 to 100 indicates eutrophic water. Under the same trophic status, the higher the index is, the more serious the eutrophication is. According to this assessment result (Table 4), water in Baiyangdian Lake is mesotrophic and eutrophicconsider the following:

(4) $$\begin{array}{c} \text{TSIM}\;\left(\text{Chl}\;a\right)=10\times \left(2.46+\frac{\text{lnChl}\;a}{\ln2.5}\right),\\ \text{TSIM}\;\left(\text{TP}\right)=10\times \left[2.46+\frac{\left(6.71+1.15\times \text{lnTP}\right)}{\ln2.5}\right],\\ \text{TSIM}\;\left(\text{SD}\right)=10\times \left[2.46+\frac{\left(3.69-1.53\times \text{lnSD}\right)}{\ln2.5}\right]. \end{array}$$

In this formula, Chl *a* is the content of chlorophyll a; SD is secchi depth; TP is total Phosphorus.

According to the dominant genus assessment, dominant phytoplankton genus observed in this survey included not only indicator species for eutrophication, such as *Chlorella *sp*.,* but also indicator species for serious eutrophication, such as *Microcystis incerta *Lemm. and *Chroomonas acuta *Uterm. with indicator species for eutrophication as the dominant species. According to these two methods, Baiyangdian Lake was at eutrophic status, which was consistent with SHEN Hui-tao's [19] research conclusion in the year 2006, in which the majority of Baiyangdian Lake was at eutrophic status and the eutrophication tended to be aggravated.

## 4. Conclusions

Eutrophication degree assessing methods can be broadly divided into two types: biological monitoring method and comprehensive indicators method. This study was carried out by combing the dominant genus assessment and the index of TSIM to assess comprehensively the eutrophication degree of Baiyangdian Lake. Generally, the species composition of phytoplankton in Baiyangdian Lake changed significantly, and the density tended to increase compared to the results of comprehensive ecological surveys in recent years. Baiyangdian Lake tended to become seriously eutrophic now. It is necessary to use different approaches to assess eutrophication due to the limitation of these approaches. Moreover, both methods for eutrophication degree assessment in this study are convenient and applicable, and it can be used to assess the impacts of organic matter pollution in other similar cases across China. The finding of this study provides necessary theoretical and data support for the control of eutrophication in Baiyangdian Lake. However, further studies are still needed on the species composition, quantity characteristics, and distribution characteristics of the phytoplankton in Baiyangdian Lake for eutrophication prevention and control and for the conservation of biodiversity.

## Figures and Tables

**Table 1:** Variations of phytoplankton density and dominant species.

Year	Number (species)	Density (×10^4^ cells/L)	Dominant species	Number (species)	Percentage %	Dominant species	Number (species)	Percentage %	Data resource
2005	152	664.4	Chlorophyta	80	52.6	Cyanophyta	28	18.4	[20]
2006	155	518.2	Chlorophyta	81	52.3	Cyanophyta	29	18.7	[20]
2009	133	2084.6	Chlorophyta	65	48.9	Cyanophyta	22	16.5	This study

**Table 2:** Correlation analysis between phytoplankton density and environmental factors of Baiyangdian Lake in spring.

	T	DO	COD	COD_Mn_	pH	TN	TP	Chla	SD	Density
T	1									
DO	0.024	1								
COD	0.760*	−0.454	1							
COD_Mn_	0.867**	−0.285	0.948**	1						
pH	0.458	0.753*	0.022	0.108	1					
TN	0.274	−0.476	0.566	0.602	−0.249	1				
TP	0.286	−0.428	0.550	0.600	−0.207	0.998**	1			
Chla	0.627	0.513	0.442	0.492	0.851**	0.057	0.089	1		
SD	−0.069	−0.212	−0.264	−0.220	−0.439	−0.382	−0.399	−0.687	1	
Density	0.644	−0.132	0.651	0.543	0.308	−0.223	−0.241	0.512	−0.067	1

*Significant correlation at the level of *P* < 0.05; **significant correlation at the level of *P* < 0.01.

**Table 3:** Correlation analysis between phytoplankton density and environmental variables of Baiyangdian Lake in summer.

	T	DO	COD	COD_Mn_	pH	TN	TP	Chla	SD	Density
T	1									
DO	−0.321	1								
COD	−0.617	0.295	1							
COD_Mn_	0.087	0.518	0.100	1						
pH	0.241	0.329	−0.070	0.484	1					
TN	−0.814*	0.472	0.555	0.455	0.107	1				
TP	−0.522	0.930**	0.424	0.423	0.267	0.645	1			
Chla	−0.454	0.930**	0.337	0.669	0.313	0.687	0.982**	1		
SD	0.339	−0.089	−0.342	−0.556	−0.364	−0.724*	−0.318	−0.622	1	
Density	−0.245	0.813*	−0.011	0.498	0.630	0.505	0.833*	0.818*	−0.357	1

*Significant correlation at the level of *P* < 0.05; **significant correlation at the level of *P* < 0.01.

**Table 4:** Assessment results of trophic status index of Baiyangdian Lake in 2009.

Item	TSIM	Trophic status
TP	77.00	Eutrophic
Chla	47.6	Mesotrophic
SD	69.24	Eutrophic
